# Perioperative and Oncological Outcomes of Percutaneous Radiofrequency Ablation versus Partial Nephrectomy for cT1a Renal Cancers: A Retrospective Study on Groups with Similar Clinical Characteristics

**DOI:** 10.3390/cancers16081528

**Published:** 2024-04-17

**Authors:** Milosz Jasinski, Przemyslaw Wisniewski, Marta Bielinska, Jerzy Siekiera, Krzysztof Kamecki, Maciej Salagierski

**Affiliations:** 1 Urology Department, Collegium Medicum, University of Zielona Góra, Zyty 28, 65-046 Zielona Góra, Poland; m.salagierski@cm.uz.zgora.pl; 2 Department of Urology, Institute of Oncology, Romanowskiej 2, 85-796 Bydgoszcz, Poland; 3 Department of Coordination of Oncological Patient Handling, The University Clinical Centre in Gdańsk, Dębinki 7, 80-952 Gdańsk, Poland

**Keywords:** radiofrequency ablation, kidney cancer, ultrasound, partial nephrectomy

## Abstract

**Simple Summary:**

Ultrasonography-guided percutaneous radiofrequency ablation is an attractive alternative treatment method for patients with small renal tumours. It has been compared to current standard—partial nephrectomy—in several studies. Most of them, however, are limited by a selection bias. In this study, we evaluated the results of ultrasonography-guided percutaneous radiofrequency ablation and partial nephrectomy in patients who, due to tumour- and patient-related factors, were most suitable for both treatment methods. The oncological results of both methods were comparable, all of recurrent or residual tumours were successfully re-treated. Percutaneous ablation was associated with significantly shorter procedure length and hospital stay, lower blood loss and analgesics used.

**Abstract:**

Over the recent years, progress in imaging techniques has led to an increased detection of kidney tumours, including small renal masses. While surgery is still the standard of care, there is a growing interest in minimally invasive methods. Ultrasound (US)-guided percutaneous ablation is particularly attractive because it is a safe and relatively simple procedure. In this study, we investigated the results of US-guided percutaneous radiofrequency ablation (RFA) and partial nephrectomy (PN) in the treatment of cT1a renal cancers. Between August 2016 and February 2022, 271 patients with renal tumours underwent percutaneous RFA as initial treatment in our institution. In the same period, 396 patients with renal tumours underwent surgical tumour excision. For the purpose of this study, only patients with confirmed renal cancer with matched age and tumour characteristics (size, location) were selected for both groups. Thus, a group of 44 PN patients and 41 RFA patients were formed with the same qualification criteria for both groups. Parameters such as procedure length, blood loss, hospital stay, analgesics used, and pre- and post-procedural serum creatinine were compared between these groups. Patients followed up with contrast-enhanced CT. There was no significant difference in age, tumour size, tumour location, and creatinine levels between these groups. All procedures were generally well tolerated. During a median follow-up of 28 months, two cases of recurrence/residual disease were found in each group. The overall survival was 100% in both groups, and all patients were disease-free at the end of observation. Percutaneous RFA was associated with a significantly shorter procedure length and hospital stay, lower blood loss, and lower analgesics used than PN. In the selected group of renal cancer patients, US-guided percutaneous RFA was associated with a shorter hospital stay, less analgesics used, and a shorter procedure length than PN, without differences in the oncological results or kidney function.

## 1. Introduction

Over the recent years, the progress in imaging techniques and wide introduction of ultrasonography (US) and computed tomography (CT) imaging has led to an increased detection of renal tumours, including small renal masses (SRM, kidney tumours smaller than 4 cm) [1,2]. While surgery is still the standard of care, the efficacy of thermal ablation (TA) in the treatment of SRMs has already been demonstrated [3,4,5,6]. Ablative techniques, such as radiofrequency ablation (RFA), were initially suggested in older patients with significant comorbidities as an alternative to partial nephrectomy (PN) due to its lower burden than surgery [2,5,6,7,8]. However, recent data suggest the efficacy of TA in all patients with tumours < 3 cm [4,5,9,10].

Recently, some studies have compared the clinical outcomes of TA versus PN [11,12,13,14,15,16]. Nevertheless, a major limitation of these studies is a selection bias, with different qualification criteria resulting in significantly different patients with different tumours being treated with TA and PN. As far as we know, there is only a single prospective, randomised study comparing percutaneous TA to PN in the treatment of SRMs published in 2023 [17].

In this study, we investigated the results of RFA and PN in the treatment of T1a renal cancers with exactly the same qualification criteria for both groups.

## 2. Materials and Methods

This retrospective observational study was approved by the institutional review board.

We analysed patients with renal tumours who underwent percutaneous RFA or surgical tumour excision as an initial treatment in our institution between August 2016 and February 2022. Recurrent lesions were not included in this study.

Medical records were retrospectively reviewed for patients’ demographics, clinical data, and procedural details. Tumour anatomic features were evaluated in pre-procedural contrast-enhanced imaging (CT or MR). For each, tumour size was measured and location in kidney was described as upper, central, or lower pole; lateral; medial-anterior; or medial-posterior. The tumour was described as exophytic (at least one-third exophytic) or non-exophytic (less than one-third).

The inclusion criteria were as follows: age under 67, functional contralateral kidney, no significant comorbidities that would be a contraindication to PN. The pre-procedural imaging was reviewed to include only patients with exophytic lesions and one of the following: not larger than 30 mm and located in central part of the kidney; not larger than 30 mm and located in the lower pole of the kidney; not larger than 25 mm. Such tumour characteristics (size, location) were previously found to be associated with the highest success rate of US-guided percutaneous TA [5].

Patients without histopathologically confirmed renal cell carcinoma (RCC) or with missing biopsy data, with inconclusive biopsy, lost from follow-up (no follow-up contrast-enhanced imaging available), or with lacking diagnostic imaging data were excluded from the study.

Patients were divided into two groups: those who had undergone percutaneous RFA and those who had undergone PN (laparoscopic or open). Parameters such as procedure length, blood loss, hospital stay, analgesics used, and pre- and post-procedural serum creatinine were compared between these groups. In this particular group of patients, the qualification either to RFA or PN was based mainly on their preference, without any specific criteria.

All patients had contrast-enhanced imaging, either CT or MR, before the procedure. All tumours undergoing RFA were biopsied, either during the ablation or before, as a separate procedure. All the pathological samples were evaluated by the same pathologists in a high-volume institution.

All ablations were performed percutaneously under US guidance in analgosedation and local anaesthesia by MJ and JS, both experienced in TA and US-guided procedures. For each procedure, Covidien Cool-tip™ RF Ablation System (Medtronic, Warszawa, Poland) was used. Ablation was performed with one probe, and the length of ablation and eventual probe repositioning were decided according to size, shape, and characteristics of the lesion. All surgical excisions were performed either laparoscopically or as an open procedure without clamping of renal vessels by different surgeons in a high-volume institution.

Patients followed up with diagnostic imaging; contrast-enhanced CT or MR was performed at 3 months, 12 months after the procedure, then yearly (RFA) or at 6 months, 12 months after the procedure, then yearly (PN). Follow-up scans were evaluated to assess the outcome. The follow-up time was calculated from the procedure to the last diagnostic imaging available. The treatment failure (local relapse) was defined as follows: in the case of percutaneous TA—the presence of enhancing tissue at the margins of the ablation volume in the first follow-up scan (residual disease) or within the ablation zone after at least one contrast-enhanced follow-up study demonstrating absence of viable tissue within the target tumour and surrounding ablation margin (local progression), in the case of PN—the presence of abnormal, enhancing tissue next to the resection zone (local recurrence).

We performed statistical analysis using Statistica 8.0 (StatSoft, Kraków, Poland) software. Differences between variables were assessed using Mann–Whitney U-test. The χ-square test was employed to evaluate differences in qualitative variables; *p* < 0.05 was considered statistically significant.

## 3. Results

During the studied period, 271 patients with renal tumours were treated with percutaneous RFA as the initial treatment in our institution. A total of 70 patients were excluded from this study. From the remaining group of 201 patients, we selected only those meeting the inclusion criteria. Thus, a group of 41 patients with ‘ideal tumours’ and who had undergone percutaneous RFA was formed.

During the same period, 396 patients with renal tumours were treated with PN (open or laparoscopic) as the initial treatment in our institution. Three hundred twenty-three patients older than 67, without functional contralateral kidney, with benign lesions, with lesions larger than 3 cm, or lost from follow-up (no follow-up contrast-enhanced imaging available) were excluded from this study. We selected a group of 44 patients with kidney tumours most suitable for percutaneous TA who had undergone PN (25 laparoscopic and 19 open). Thus, a group of 44 PN patients and 41 RFA patients were selected.

There was no significant difference in age, tumour size, tumour location, or creatinine level between these groups. The characteristics of studied groups are presented in Table 1.

The procedures were generally well tolerated. We registered four Clavien-Dindo grade I complications: three cases of fever (one in RFA, one in laparoscopic PN, one in open PN) and one case of wound haematoma (open PN). We also registered three Clavien-Dindo grade II complications: one grade II bowel injury treated conservatively in the RFA group and two cases of blood loss requiring transfusion in the PN group (one in laparoscopic, one in open).

The mean follow-up time was 29 months, and the median was 28 months (range 3–71 months).

During follow-up, one case of residual disease (enhancement in CT/MR 3 months after the procedure) and one case of local progression later than 3 months despite initially complete ablation (no enhancement in CT/MR 3 months after procedure) were found in the RFA group. These cases were treated with repeated thermal ablation sessions (one additional procedure). Two cases of local recurrence were found in the PN group; one was treated with percutaneous RFA and one with surgical resection.

The overall survival was 100% in both groups, and all patients were disease-free at the end of observation.

There was a significant difference between PN and RFA in procedure length, hospital stay, blood loss, and analgesics used (Table 2). Blood loss during percutaneous ablation is negligible. There was no ischaemia in both groups.

Interestingly, when laparoscopic and open PN were compared, there were significant differences in blood loss and hospital stay but not in the analgesics used. Patients undergoing laparoscopic PN were younger, but the difference in tumour size was not significant (Table 3).

There were two conversions from laparoscopic to open—one because of bleeding and one because of other technical difficulties. Even if compared to only the laparoscopic PN group, the percutaneous RFA group still has significantly lower blood loss, a shorter procedure length, a shorter hospital stay, and less analgesics used (Table 4).

## 4. Discussion

PN remains the gold standard in the management of SRMs with established short- and long-term outcomes [13,18,19,20]. It is a well-known, thoroughly studied and described treatment method with known and established indications. It can be performed both as an open or endoscopic (laparoscopic or robot-assisted) procedure. US- or CT-guided percutaneous TA has been developed over the last decades and is emerging as an alternative treatment with curative intent for SRMs [2,3,5,13,18,21,22]. It can be performed under analgosedation or general anaesthesia and is relatively well tolerated, which makes it a viable option for patients with comorbidities or who are unfit for surgery [2,4,5,6]. Indeed, the European Association of Urology recommends thermal ablation as an alternative for frail and/or comorbid patients with small renal masses [18].

Over the last few years, several studies comparing thermal ablation to PN have been published [13,14]. Although there is a general agreement that thermal ablation is a safe and effective treatment, the details are not so consistent. While some authors reported equivalent outcomes [22,23,24], a similar overall survival and cancer-specific survival [25], and no statistically significant difference in local recurrence [12,24], others reported higher recurrence rates [11,26,27], and/or worse overall survival in patients treated with thermal ablation compared with surgery [13]. A meta-analysis from 2016 reported inferior local oncologic control in patients treated with TA compared with patients treated with PN; however, with retreatment, RFA was no longer inferior [28]. These conflicting—to some degree—results may be partially due to the fact that most of these studies are of somewhat limited quality and restricted by a significant selection bias [11,13,14,29]. The inclusion criteria differ, and there is a tendency to perform PN in younger, fit patients and thermal ablation in older and comorbid ones. Some studies also include SRMs with benign histopathology. As far as we know, there is no proper, randomised prospective trial comparing percutaneous thermal ablation to PN. There is a propensity score-matched analysis comparing PN to percutaneous ablation [11]. While that study is free of selection bias, it still includes patients with benign histopathology and non-diagnostic biopsy.

There also is another possible aspect of bias: although the influence of patient-related factors (age, comorbidities, etc.) is generally recognised, the significance of tumour-related factors is much less discussed. Not all SRMs are equal, and there is a degree of ‘treatment difficulty’ related to tumour size and its location in the kidney [5]. This is rather well described for PN, with scores such as RENAL and PADUA, but much less for percutaneous ablation [5,10,30]. The scores developed for PN are not necessarily suitable for ablation and vice versa, with tumour complexity differently influencing the risk of recurrence and complications for PN and ablation. Therefore, even if all patient-related factors are matched, there is still a possibility of bias related to tumour complexity. It is difficult to properly estimate this issue, with many studies not reporting tumour complexity or using only scores developed for PN.

To reduce the possible bias associated with patient selection, we decided to compare the treatment results in patients who were both good candidates for surgery and US-guided percutaneous ablation. To ensure uniform selection criteria, we excluded all patients who could be poor candidates for surgery due to age and/or comorbidities. Furthermore, the pre-procedural diagnostic imaging was reviewed by MJ, JS (experienced in TA and PN), and PW (experienced in PN and laparoscopic PN), and all patients who could be suboptimal for RFA due to tumour size and/or location were excluded. This allowed us to have no significant differences in characteristics of patients and tumours between groups and ensure that the only significant selection criteria were patients’ and surgeons’ preference. Finally, we excluded all lesions with benign or unconfirmed histopathology.

Another issue that should be considered is the definition and criteria of cancer relapse. The diagnosis of local recurrence both after PN and TA is based mainly on diagnostic imaging. We do not routinely biopsy local recurrences due to the high rate of non-diagnostic biopsies and difficulties in interpretation what is a negative result in this situation. Therefore, we had a histopathological confirmation of local recurrence only in patients treated with surgical resection. There are, however, some significant differences between PN and TA. PN in most cases (all in this study) is a macroscopically complete resection, and the local recurrence is the presence of abnormal, enhancing tissue next to the resection zone. In the case of percutaneous TA, the definition of recurrence is more complex [27]. As the tumour tissue is not resected and it may be sometimes difficult to determine the macroscopic completeness of the treatment, residual disease (the presence of enhancing tissue in the ablation volume in the first follow-up scan) is more common than it is after PN. It is also possible to find enhancing tissue within the ablation zone after a previous contrast-enhanced study demonstrating no enhancement (local progression). Furthermore, currently, there is no consensus of surveillance intervals after TA [27].

Several of our findings are remarkable. First, our study is different from most studies comparing the ablation of renal masses to PN in the aspect of patient selection. Because of the above-mentioned selection criteria, patients in our study are significantly younger than patients included in most other studies—at least in the ablation arms [12,13,14,16]. We have also excluded patients with significant comorbidities, often included in the ablation groups in other studies [12,13,14,16,29].

Second, due to these selection criteria, there was no other-cause mortality; all patients remained alive through the observation period. The oncological results were also good, with no systemic progression and four cases of local relapse successfully treated with repeated RFA or surgical resection. Such good oncological results, however, could be expected for relatively small, exophytic, and easy-to-manage tumours. In contrast to many other studies, there was no significant difference in local relapses between the RFA (4.8%) and PN (4.5%) group. This can be explained by the fact that in this study, we included only the tumours most suitable for percutaneous RFA, as described previously [5].

Third, while both procedures were well tolerated, with only 3 cases of grade II complications (3.5%) and no Clavien-Dindo grade ≥ III complications, there were significant differences in procedure length, hospital stay, blood loss, and analgesics used in favour of RFA [Table 2]. Interestingly, we observed no deterioration of kidney function, even in the PN group. Tumour complexity and warm ischaemia were found to be associated with renal function loss [31]. Perhaps our results may be explained by the fact that all lesions were low-complexity ones, and all procedures were performed without ischaemia.

We also observed that laparoscopic PNs were associated with significantly lower blood loss and a shorter hospital stay than open procedures, which is consistent with other studies [4,32]. Thus, it may be more appropriate to compare the burden of percutaneous ablation to laparoscopic or robotic PN. However, even if compared to only the laparoscopic PN group, the percutaneous RFA group still has significantly lower blood loss, a shorter hospital stay, and less analgesics used. This is consistent with the results of recent studies comparing robot-assisted PN (RAPN) to percutaneous ablation in challenging situations (solitary kidney, endophytic tumour) [33,34]. In these studies, authors found percutaneous ablation to be associated with a shorter stay, a shorter procedure length, and less complications than RAPN. There were, however, significant differences in tumour characteristics between RAPN and ablation patients.

Both percutaneous RFA and PN have strengths and weaknesses. Percutaneous ablation is associated with significantly lower morbidity, which may be particularly attractive for older patients with comorbidities who have an increased risk of serious surgery-related complications. It also offers potentially better kidney function preservation—but possibly not significantly in the case of the smallest, ‘easiest’ tumours. PN, on the other hand, has an oncological effect less sensitive to factors associated with tumour size and location, with a wider range of SRMs that could be treated without the increased risk of relapse. With the advancement in surgical techniques, the indications for RAPN are expanding to even more challenging tumours. Recently, RAPN was reported to offer encouraging results in the case of challenging perihilar masses [35].

While this study does not prove that percutaneous ablation is an equivalent of PN in all SRMs, it proves that percutaneous RFA may offer reduced morbidity without sacrificing oncological results in a selected subgroup of SRMs. This study was focused on the least complex tumours, in which we could expect good oncological results both for TA and PN, without a significant difference in this aspect. The reduced morbidity and shorter hospital stay was, however, in favour of TA. It may lead to a conclusion that there are kidney tumours for which the percutaneous TA could be the preferred treatment. Further effort should be made to clearly identify the tumour-related qualification criteria for percutaneous ablation so that this method could also be offered to some of the younger and healthy patients without the increased risk of disease recurrence.

In order to provide better quality of evidence concerning TA as a treatment for SRMs, some aspects should be considered when designing new studies. First, to avoid patient-related bias, studies should preferably be prospective randomised trials with clear inclusion and exclusion criteria or at least have uniform qualification criteria for all groups. Second, if a study is not a randomised trial, the groups should be matched for patient-related factors, such as age and comorbidities. Third, more attention should be given to the influence of the tumour-related factors. To avoid bias in non-randomised trials, the groups should be matched for tumour size and location. All trials, even the randomised ones, should report tumour size and location in more details, as certain tumour-related factors may favour one of the treatment methods. Finally, groups should be matched for or not include benign lesions; preferably, all lesions should be biopsied before inclusion, and only patients with a diagnostic biopsy result should be included.

We have made an effort to overcome several limitations associated with many studies comparing renal tumour ablation to PN: we used uniform inclusion criteria, we only included confirmed RCC, and all patients were treated in the same period in one institution. Despite these strengths, this study is not free from limitations. First, it is still a retrospective study, and despite the uniform inclusion criteria, differences between groups and some form of selection bias may still exist. Second, the PN group was not uniform; it included both open and laparoscopic procedures, with some significant differences between them. Third, the median follow-up time was 28 months, which may be insufficient to fully assess the long-term outcomes. On the other hand, most residual disease/recurrences are detected within the first 2 years [36]. Finally, many patients were excluded because of missing data or being lost from follow-up.

## 5. Conclusions

In conclusion, in a selected subgroup of RCC patients, percutaneous RFA was associated with a significantly shorter procedure length and hospital stay, lower blood loss, and less analgesics used than PN, but no difference in oncological results nor kidney function preservation was observed. It must be stressed that these results should not be extrapolated to all SRMs, as the tumours included in this study do not reflect the entirety of SRMs. There is a need for a prospective randomised trial to solidify these findings and better define the role of percutaneous TA in the treatment of SRMs.

## Figures and Tables

**Table 1:** Characteristics of patients and tumours.

	PN	RFA	*p*
*n*	44	41	
Age (mean ± SD) [y]	55.2 ± 9.0	56.3 ± 9.8	NS
Diameter (mean ± SD) [mm]	22.23 ± 4.6	22.27 ± 4.5	NS
Diameter (%):			*p* = 0.449
≤25 mm	68.2	75.6	
25–30 mm	31.8	24.4	
Location (%):			
Upper pole	4.5	12.2	*p =* 0.196
Central	52.3	53.6	*p =* 0.904
Lower pole	43.2	34.2	*p* = 0.395
Laterality (%):			
Lateral	84.1	78.1	*p* = 0.479
Medial posterior	4.5	14.6	*p* = 0.110
Medial anterior	11.4	7.3	*p* = 0.517
Creatinine before (mean ± SD) [µmol/L]	75.4 ± 15.7	77.2 ± 18.2	NS
Creatinine after (mean ± SD) [µmol/L]	78.1 ± 16.3	77.0 ± 18.6	NS

PN—partial nephrectomy, RFA—radiofrequency ablation.

**Table 2:** PN vs. RFA.

	PN	RFA	*p*
*n*	44	41	
Procedure length (mean ± SD) [min] median (range)	103 ± 25 95 (70–180)	25 ± 7 20 (20–40)	*p* < 0.001
Hospital stay (mean ± SD) [days] median (range)	5.04 ± 0.67 5 (4–7)	2.12 ± 0.94 2 (1–8)	*p* < 0.001
Blood loss (mean ± SD) [mL]	209 ± 153	0	*p* < 0.001
Opiates (%)	82%	7%	*p* < 0.001
Oxycodone/morphine (mean ± SD) [mg] median (range)	9.19 ± 7.66 9 (0–30)	0.36 ± 1.30 0 (0–5)	*p* < 0.001
Paracetamol (mean ± SD) [g] median (range)	5.52 ± 1.64 5 (2–8)	1.54 ± 1.04 2 (0–4)	*p* < 0.001
Metamizole (mean ± SD) [g] median (range)	2.14 ± 2.04 2 (0–6)	0.24 ± 0.48 0 (0–2)	*p* < 0.001
Ketoprofen (mean ± SD) [mg] median (range)	20.45 ± 59.66 0 (0–300)	9.75 ± 27.54 0 (0–100)	*p* = 0.751

PN—partial nephrectomy, RFA—radiofrequency ablation.

**Table 3:** Laparoscopic PN vs. open PN.

	PN Laparoscopic	PN Open	*p*
*n*	25	19 *	
Age (mean ± SD) [y]	52.6 ± 8.6	58.7 ± 9.1	*p* = 0.029
Diameter (mean ± SD) [mm]	21.12 ± 4.4	23.68 ± 4.7	*p* = 0.068
Procedure length (mean ± SD) [min] median (range)	105 ± 29 100 (70–180)	101 ± 17 90 (80–130)	NS
Hospital stay (mean ± SD) [days] median (range)	4.72 ± 0.54 5 (4–6)	5.45 ± 0.61 5 (4–7)	*p* < 0.001
Blood loss (mean ± SD) [mL]	168 ± 109	263 ± 189	*p* = 0.011
Opiates (%)	80%	84%	NS
Oxycodone/morphine (mean ± SD) [mg] median (range)	8.32 ± 7.01 6 (0–25)	10.15 ± 8.71 9 (0–30)	NS
Paracetamol (mean ± SD) [g] median (range)	5.24 ± 1.81 6 (2–9)	5.89 ± 1.41 6 (3–8)	NS
Metamizole (mean ± SD) [g] median (range)	2.48 ± 2.02 3 (0–6)	1.68 ± 2.08 1 (0–6)	NS
Ketoprofen (mean ± SD) [mg] median (range)	26.00 ± 69.40 0 (0–300)	13.16 ± 46.67 0 (0–200)	NS

* 2 conversions, laparoscopic to open, were included in the open PN group. PN—partial nephrectomy.

**Table 4:** Laparoscopic PN vs. RFA.

	PN Laparoscopic	RFA	*p*
*n*	25	41	
Procedure length (mean ± SD) [min] median (range)	105 ± 29 100 (70–180)	25 ± 7 20 (20–40)	*p* < 0.001
Hospital stay (mean ± SD) [days] median (range)	4.72 ± 0.54 5 (4–6)	2.12 ± 0.94 2 (1–8)	*p* < 0.001
Blood loss (mean ± SD) [mL]	168 ± 109	0	*p* < 0.001
Opiates (%)	80%	7%	*p* < 0.001
Oxycodone/morphine (mean ± SD) [mg] median (range)	8.32 ± 7.01 6 (0–25)	0.36 ± 1.30 0 (0–5)	*p* < 0.001
Paracetamol (mean ± SD) [g] median (range)	5.24 ± 1.81 6 (2–9)	1.54 ± 1.04 2 (0–4)	*p* < 0.001
Metamizole (mean ± SD) [g] median (range)	2.48 ± 2.02 3 (0–6)	0.24 ± 0.48 0 (0–2)	*p* < 0.001
Ketoprofen (mean ± SD) [mg] median (range)	26.00 ± 69.40 0 (0–300)	9.75 ± 27.54 0 (0–100)	*p* = 0.179

PN—partial nephrectomy, RFA—radiofrequency ablation.

## Data Availability

Data generated or analysed during this study are included in this article. More detailed data are not publicly available due to their containing information that could compromise the privacy of patients. Further enquiries can be directed to the corresponding author.
